# Angiotension II directly bind P2X7 receptor to induce myocardial ferroptosis and remodeling by activating human antigen R

**DOI:** 10.1016/j.redox.2024.103154

**Published:** 2024-04-09

**Authors:** Xin Zhong, Kangwei Wang, Yonghua Wang, Luya Wang, Sudan Wang, Weijian Huang, Zhuyin Jia, Shan-Shan Dai, Zhouqing Huang

**Affiliations:** a Department of Cardiology and the Key Laboratory of Cardiovascular Disease of Wenzhou, The First Affiliated Hospital of WenZhou Medical University, WenZhou, ZheJiang, China; b Department of Physical Education, WenZhou Medical University, WenZhou, ZheJiang, China; c Department of Respiratory, Wenzhou People's Hospital of Zhejiang Province, WenZhou, ZheJiang, China; d Department of Cardiology, Wenzhou Central Hospital, The Second Affiliated Hospital of Shanghai University, Wenzhou, Zhejiang, China; e Department of Emergency, The First Affiliated Hospital of Wenzhou Medical University, WenZhou, Zhejiang, China

**Keywords:** Cardiac remodeling, Angiotensin II, Ferroptosis in myocytes, P2X7R, Human antigen R, Mutagenesis

## Abstract

Continuous remodeling of the heart can result in adverse events such as reduced myocardial function and heart failure. Available evidence indicates that ferroptosis is a key process in the emergence of cardiac disease. P2 family purinergic receptor P2X7 receptor (P2X7R) activation plays a crucial role in numerous aspects of cardiovascular disease. The aim of this study was to elucidate any potential interactions between P2X7R and ferroptosis in cardiac remodeling stimulated by angiotensin II (Ang II), and P2X7R knockout mice were utilized to explore the role of P2X7R and elucidate its underlying mechanism through molecular biological methods. Ferroptosis is involved in cardiac remodeling, and P2X7R deficiency significantly alleviates cardiac dysfunction, remodeling, and ferroptosis induced by Ang II. Mechanistically, Ang II interacts with P2X7R directly, and LYS-66 and MET-212 in the in the ATP binding pocket form a binding complex with Ang II. P2X7R blockade influences HuR-targeted GPX4 and HO-1 mRNA stability by affecting the shuttling of HuR from the nucleus to the cytoplasm and its expression. These results suggest that focusing on P2X7R could be a possible therapeutic approach for the management of hypertensive heart failure.

## Graphical abstract

**Figure:**
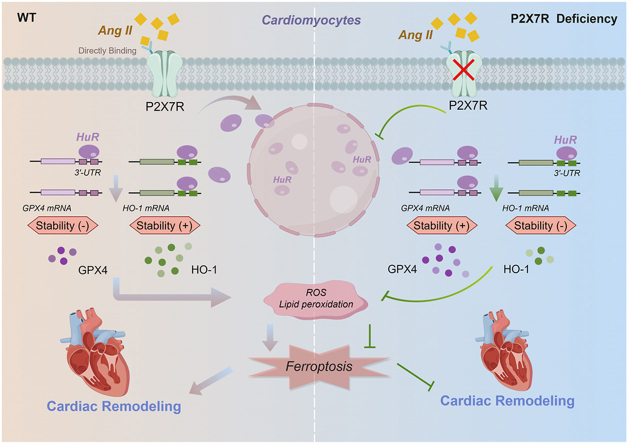

## 1 Introduction

Cardiac remodeling typically presents with cardiomyocyte hypertrophy, myocyte loss, and fibrosis. Sustained cardiac remodeling can result in adverse events, such as decreased myocardial function and heart failure; however, the remodeling process, as a compensatory mechanism, initially confers protection to the heart [1,2]. Common causes of pathological cardiac remodeling include mechanical interference, oxidative stress, inflammation, and neurohumoral activation (e.g., Ang II) [[2], [3], [4]]. Due to the adverse outcomes of persistent cardiac hypertrophy, it is necessary to understand the molecular processes driving cardiac remodeling and to discover innovative targets for preventing unfavorable cardiac remodeling and heart failure.

Although programmed cell death is essential for cellular growth, the death of myocardial cells is detrimental due to their irreversible differentiation. Cell death results in myocyte loss and compromises cardiac contractility. Consequently, this phenomenon initiates adverse cardiac remodeling, which culminates in impaired cardiac function and eventual heart failure [[5], [6], [7]]. Therefore, strategies focusing on cellular death represent a prospective therapeutic approach for mitigating cardiac remodeling. Recent studies have revealed a plethora of cellular demise mechanisms, such as apoptosis, necroptosis, necrosis mediated by mitochondria, pyroptosis, ferroptosis, and autophagic cell death [5]. Among these, ferroptosis is a recently discovered type of cell death that involves iron and lipid peroxidation. It was initially reported in 2012 [8] and has been found to participate in various pathogenic processes, including hepatocellular carcinoma and renal I/R injury [[9], [10], [11]]. Additionally, it contributes to the prevention of atherosclerosis [12]. Some recent studies have indicated that ferroptosis is a crucial type of cell death in cardiomyocytes and is a key mechanism in the occurrence of doxorubicin-induced cardiotoxicity and cardiac I/R injury [13,14]. In addition, Zhong and colleagues [15] reported that elabela suppressed ferroptosis in cardiac microvascular endothelial cells, leading to the alleviation of cardiac fibrosis and hypertrophy. Nevertheless, it remains unclear how ferroptosis of cardiomyocytes contributes to the cardiac remodeling induced by Ang II.

The P2X7 receptor, a member of the purinergic receptor family, was cloned in the mid-1990s and consists of 595 amino acids [16]. P2X7R is expressed in many cell types, including microglia, nerve cells, mast cells, cardiomyocytes and monocytes [17]. P2X7R activation plays crucial roles in diverse aspects of cardiovascular diseases, especially by regulating inflammation to promote the release of proinflammatory cytokines and ROS generation, contributing to atherosclerosis, myocardial ischemic injury and myocardial infarction development [18]. In addition, P2X7R deficiency mitigates cardiac apoptosis and fibrosis in diabetic cardiomyopathy [19]. These data demonstrate that P2X7R activation is detrimental to the vasculature or heart, suggesting that P2X7R has the potential to serve as an effective therapeutic target to avoid cardiac injury. However, except inflammation, the pivotal effects of pathological cardiac ferroptosis remain largely uncharacterized.

In this particular study, we demonstrated that Ang II stimulation in mice significantly promoted cardiac ferroptosis and P2X7R expression. Additionally, we examined the impact of ferroptosis suppression or P2X7R deletion on cardiac remodeling in the hearts of mice given Ang II injections and explored the downstream target/mechanism of P2X7R in regulating ferroptosis of myocytes.

## 2 Materials and methods

### 2.1 Animal experiments

The guidelines for the care and use of laboratory animals published by the National Institutes of Health (USA) were strictly followed when handling and caring for any experimental animals in this study. Furthermore, the Animal Ethics Committee at the First Affiliated Hospital of Wenzhou Medical University (Approval No. 2021-0262) rigorously reviewed and approved all the animal research. Global germline male P2X7R knockout (P2X7R–KO) mice and male C57BL/6 wild-type mice were acquired from GemPharmatech Co., Ltd., Nanjing, China. All mice were housed in an SPF barrier system, fed irradiated rodent food, and given sterile water in a constant environment of 22 °C, 50 % humidity, and 12 h of light and darkness per day.

Cardiac remodeling was achieved in 8 ∼ 12-week-old mice weighing 24–26 g through chronic subcutaneous infusion of angiotensin II (#A107852, Aladdin, 1 μg/kg/min) with ALZET® Osmotic Pumps (Model 1004) for four weeks. The normal group was injected with saline for 4 weeks. For in vivo drug treatments, ferrostatin-1 (Fer-1) (#S7243, Selleck, 1 mg/kg/day), a ferroptosis inhibitor, was intraperitoneally injected into mice every day, and deferoxamine mesylate (DFO) (#S5742, Selleck, 30 mg/kg/day), an iron chelator, was intraperitoneally injected into mice three days a week. After four weeks, all the mice were humanely allowed to sleep under sodium pentobarbital anesthesia, and their serum and hearts were collected.

### 2.2 Histological analysis, transmission electron microscopy and rhodamine phalloidin staining

Heart tissue fixed in 4 % paraformaldehyde was embedded in paraffin after a series of gradient dehydrations and then sectioned at a thickness of 5 μm. After a series of dewaxing procedures, hematoxylin and eosin (H&E) or wheat germ agglutinin (WGA, Genetex, GTX01502) was used to stain the various sections. The amount of collagen deposition was determined by Masson's trichrome (G1340, Solarbio, China) or Sirius Red staining (G1472, Solarbio, China). An anti-4-hydroxynonenal antibody (MAB3249, R&D Systems) was used for immunohistochemical staining of heart sections according to our previously described procedures [20]. The pathological sections were imaged using an upright microscope (400 × magnification; Leica, Germany) and subsequently subjected to histopathological evaluation.

For transmission electron microscopy (TEM), the myocardial tissues were fixed with 2.5 % glutaraldehyde for 24 h at 4 °C, followed by rinsing with phosphate-buffered saline (PBS) three times. Subsequently, the tissues were further fixed with 1 % osmic acid at room temperature in the dark for 1 h. After triple rinsing with double distilled water, they were stained with a solution containing 1 % uranyl acetate at room temperature in the dark for 1.5 h. The tissues were added to a 50 % embedding solution and incubated for 2 h at 37 °C after acetone gradient dehydration. Then, they were incubated with an 80 % embedding solution overnight at 37 °C. The next day, pure embedding solution was used to immerse the tissue for 2 h at 45 °C and then for 48 h at 65 °C. The tissue was precisely divided into ultrathin sections measuring approximately 70–80 nm, followed by imaging with a Hitachi H-7500 transmission electron microscope.

To test the size of the cells, rhodamine phalloidin staining (CA1610, Solarbio, China) was performed following the manufacturer's instructions. The cells were observed using an inverted fluorescence microscope (400 × amplification; Nikon, Japan).

### 2.3 Cardiac function

The cardiac remodeling caused by Ang II persisted for a period of 4 weeks. Cardiac function was assessed one day prior to 4 weeks via a high-resolution small animal ultrasound imaging system (Vevo-3100). Anesthesia was noninvasively administered and maintained in mice using isoflurane (gas flow rate generally set at 2 L/min, with an oxygen concentration ranging from 0.5 % to 1 %) through a small animal gas anesthesia machine. The specific operational procedure of the machine was previously described [21]. Echocardiography data analysis was conducted using Vevo LAB3.1.1 software, with all cardiac function parameters automatically calculated by the software.

### 2.4 Culture and treatment of cardiomyocytes

We isolated primary cardiomyocytes from Sprague‒Dawley rat heart tissue using previously described procedures [22]. H9c2 cardiomyocytes were acquired from the National Collection of Authenticated Cell Cultures (Shanghai, China). All cells were cultured with DMEM containing glucose (Gibco, USA, 4.5 g/L), and 10 % fetal bovine serum (Gibco, USA) and 1 % penicillin and streptomycin solution were added at 37 °C in a 5 % CO_2_ incubator. Cardiomyocytes were pretreated with A-438079 (an inhibitor of P2X7R) from Selleck (#S7705, 10 μM), deferoxamine mesylate from Selleck (#S5742, 50 or 100 μM), or ferrostatin-1 from Selleck (#S7243, 1 or 5 μM) for 2 h before stimulation with Ang II (1 μM).

### 2.5 Cell siRNA transfection

In H9c2 cells, siRNA was used to silence P2X7R and HuR (also known as Elavl1, human antigen R). Rat P2X7R siRNA (5ʹ-CGCUGUCAACCCAAAUACA-3ʹ) and rat HuR siRNA (5ʹ-CCGAGTTTCTGAGACCATT-3ʹ) were acquired from RiboBio (Guangzhou, China). RiboBio transfection reagents were used for all transfections following the protocol provided by the manufacturer. P2X7R and HuR expression levels were assessed via Western blotting and RT‒PCR analysis after 24 h of transfection.

### 2.6 Immunofluorescence staining

Paraffinized and rehydrated heart slices were microwaved with citric acid buffer to recover antigens. To limit endogenous peroxidase activity, the slides were treated with 5 % bovine serum albumin (BSA) for 30 min at room temperature after being incubated with a 3 % H2O2 solution for 15 min. The slides were then incubated with primary antibodies against P2X7R (rabbit, 1:200, #APR-004, Alomone Labs), alpha-cardiac actin (mouse, 1:200, M1206-1, Huabio) and vimentin (mouse, 1:200, EM0401, Huabio) overnight at 4 °C. On the following day, the slides were incubated with iFluor™ 594-conjugated goat anti-rabbit IgG goat polyclonal antibody (1:200, HA1122, Huabio) and iFluor™ 488-conjugated goat anti-mouse IgG goat polyclonal antibody (1:200, HA1125, Huabio) for 1 h at room temperature. Finally, the slides were stained with 20 μl of DAPI Fluoromount-G™ (36308ES11, Yeasen Biotechnology) for nuclear staining.

After receiving the appropriate treatment, H9c2 cells were rinsed three times in PBS prior to being immobilized for 15 min at ambient temperature in 4 % paraformaldehyde. Afterward, the cells were treated with 0.5 % Triton X-100 for 15 min for clearing, followed by a 30-min blocking step at room temperature using 5 % BSA in PBS. The cells were incubated overnight at 4 °C with primary antibodies targeting HuR (1:200, ET1705-81, Huabio) or P2X7R (1:200, ER1919-21, Huabio). The next day, the cells were placed in an incubator for 1 h at ambient temperature with iFluorTM 488-linked goat anti-rabbit IgG. A Nikon fluorescence microscope was used to capture all of the fluorescence images.

### 2.7 Measurement of intracellular ROS

Following the manufacturer's instructions, the Lipid Peroxidation MDA Assay Kit (#S0131) obtained from Beyotime was used to detect the malondialdehyde (MDA) content, the Total Superoxide Dismutase Assay Kit with WST-8 (#S0101S) purchased from Beyotime was used to assess total superoxide dismutase (SOD) activity, and the GSH and GSSG Assay Kit (#S0053) purchased from Beyotime was used to test reduced glutathione (GSH) levels in cells.

### 2.8 Western blot analysis

Heart tissues and H9c2 cells were lysed via cell lysis buffer, followed by centrifugation at 12,000 rpm for 10 min at 4 °C to obtain the supernatants. A BCA Protein Assay Kit (PC0020, Solarbio) was used to assess the protein concentration in the histiocyte lysates. The corresponding protein samples were added to each well for 10 % or 12 % SDS‒PAGE and separated at 80 or 120 V. Then, the protein was transferred to a nitrocellulose membrane (#HATF00010, Millipore) with a current of 300 mA for 60–90 min. Subsequently, 5 % nonfat milk was used to block the NC membranes at room temperature for 1.5 h, followed by incubation with primary antibodies against ANP (sc-515701, Santa Cruz Biotechnology), COL-1 (sc293182, Santa Cruz Biotechnology), P2X7R (ER1919-21, Huabio), TGF-β (ER31210, Huabio), HuR (ET1705-81, Huabio), GPX4 (ET1706-45, Huabio), β-MYHC (ab50967, Abcam), MMP9 (ab228402, Abcam), and HO-1 (ab68477, Abcam) overnight at 4 °C. The following day, the membranes were incubated with HRP-labeled goat anti-rabbit or anti-mouse IgG (A0208 or A0216, Beyotime) at room temperature for 1 h. Meilunbio® fg supersensitive ECL luminescence reagent (MA0186, Meilunbio) was used to detect protein bands, and ImageJ analysis software was used for protein quantification.

### 2.9 Quantitative real-time polymerase chain reaction (qPCR)

H9c2 cells and heart tissues were lysed with TRIzol (Thermo Fisher) for RNA isolation. The reverse transcription reagents used in this study were procured from Vazyme Biotech (Nanjing, China). SYBR Green fluorescent quantitative reverse transcription reagent was used for reverse transcription and quantitative PCR. Sangon Biotech (Shanghai, China) designed and produced the primers utilized in the investigation. All primer sequences are listed in Supplementary Table S1. GAPDH was used to standardize the levels of other mRNAs.

### 2.10 Assessment of mitochondrial membrane potential (MMP)

The MMP of H9c2 cells was measured using a mitochondrial membrane potential assay kit with JC-1 (#C2006, Beyotime). In six-well plates, cells were seeded and then stimulated for 24 h with Ang II (1 μM) either with or without a 2-h pretreatment with A438079 (10 μM), followed by 20 min of incubation in JC-1 solution at 37 °C. Changes in the MMP are shown by an increase in green fluorescence and a decrease in red fluorescence in the cells.

### 2.11 Measurement of ferrous iron

The levels of ferrous iron in the serum and heart tissue were detected by a Ferrous Iron Colorimetric Assay Kit (*E*-BC-K772-M, Elabscience). The levels of ferrous iron in the cells were measured with a Cell Ferrous Iron Colorimetric Assay Kit (*E*-BC-K881-M, Elabscience). All procedures and calculations were performed following the manufacturer's instructions.

### 2.12 Enzyme-linked immunosorbent assay (ELISA)

To determine the concentration of Ang II in the serum, a human Ang II ELISA kit (E-EL-H0326c, Elabscience) was used. One hundred microliters of serum was used for the measurement. All procedures and ultimate evaluations were conducted in accordance with the guidelines provided by the manufacturer.

### 2.13 Determination of mRNA stability

Actinomycin D (#S8964, Selleck) was used to detect mRNA stability via an mRNA decay assay. H9c2 cells subjected to the corresponding treatments were incubated with actinomycin D (4 μM) for 0, 1, 2, 4, 6, or 8 h. Then, total mRNA was extracted from the cells with TRIzol, and the mRNA levels of HO-1 and GPX4 were measured via qPCR.

### 2.14 RNA-binding protein immunoprecipitation (RIP)

The RIP experiment was performed with an RNA-Binding Protein Immunoprecipitation Kit (17–700, Millipore, USA). The antibodies used in the RIP experiment included HuR (ET1705-81, Huabio, Hangzhou) and anti-rabbit IgG as a negative control. The mRNA expression levels of HO-1 and GPX4 were determined by qPCR.

### 2.15 Ang II-P2X7R binding assays

The interaction between Ang II and P2X7R was detected using biolayer interferometry (BLI) with Octet R8. Biotinylated Ang II (bio-Ang II) was specifically captured by an SA chip, and after reaching a signal of 0.8 nm, it was bound to different concentrations of the P2X7R protein. Bio-Ang II was diluted to 1 μg/ml in a total volume of 1200 μL using 1 × PBS and cured for 60 s. The P2X7R protein was diluted to the highest concentration of 1 μM using SD buffer and then further diluted to five concentrations: 1 μM, 0.5 μM, 0.25 μM and 0.0625 μM. The resulting data were processed using ForteBio Data Analysis 12.0 software for alignment.

We used biotinylated Ang II (Bio-Ang II), which was created by Beijing Fanbo Biological Chemical Co., Ltd., and streptavidin magnetic beads (HY–K0208, MedChemExpress) for the pull-down assay. In brief, streptavidin-agarose beads (20 μL) were combined with Bio-Ang II (100 μL, 1 mM), and the mixture was incubated at room temperature for 30 min.

After the bead-Ang II complex was washed with PBS, 5 % BSA (500 μL) and biotin (1 μL, 100 mM) were mixed and incubated for 60 min at room temperature to minimize nonspecific binding. Untreated beads (Ctrl), unbiotinylated Ang II (Ang II), and biotin alone (Bio) were used as controls. Lysates derived from the hearts of mice treated with sham or Ang II or from primary cardiomyocytes stimulated with Ang II were subsequently incubated with the bead-Ang II complex at 4 °C for 24 h under mild agitation. The mixture was subjected to three rounds of centrifugation and washing. The eluent was mixed with 5x loading buffer and boiled for Western blotting. The total lysates were used as a control for the experimental input.

### 2.16 Molecular docking

Using the molecular docking tool Discovery Studio 4.5, we determined how the chemical Ang II interacted with the 3D structure of P2RX7 (PDB code: 6U9 W). The protein structure was prepared using LibDock software. Prior to docking, both the protein structures and ligands were preprocessed via the Prepare Protein and Prepare Ligands modules. The conformation with the highest docking score was selected for analysis of the receptor‒ligand interaction.

### 2.17 Statistical analysis

All the data were analyzed using GraphPad Prism 8.0 and are expressed as the mean and SEM. For comparisons involving more than two groups of data, two-way ANOVA followed by Tukey's correction was employed, while the *t*-test was used for comparisons between two groups of data. A P value of 0.05 was considered to indicate statistical significance.

## 3 Results

### 3.1 Increased ferroptosis and ROS in the myocardium of Ang II-induced mice

**Fig. 1:**
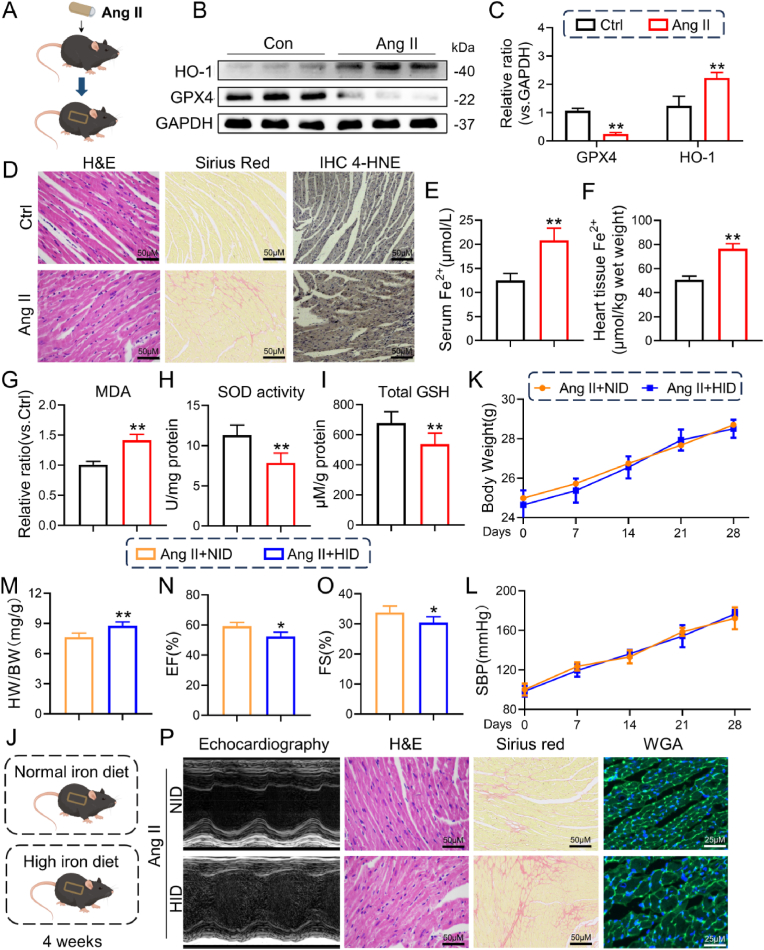
Increased ferroptosis and ROS in the myocardium of Ang II-induced mice. **A** C57BL/6 mice were continuously subcutaneously infused with Ang II using ALZET® Osmotic Pumps (Model 1004) for 4 weeks. **B** Representative Western blot analysis of HO-1 and GPX4 levels in the myocardium of mice. GAPDH was used as a loading control. **C** Densitometric quantification of immunoblots in A (n = 6; *versus the Ctrl group; *P < 0.05, **P < 0.01). **D** Representative images of H&E staining, Sirius red staining and 4-HNE immunoreactivity in the myocardium of mice. **E** and **F** The levels of Fe^2+^ in serum (**E**) and heart tissue. **G-I** Representative MDA content, SOD activity and GSH levels in the myocardium of mice. **J** C57BL/6 mice were fed a normal-iron diet (NID) or a high-iron diet (HID) and infused with Ang II for 4 weeks. **K** and **M** Body weight (**K**) and SBP (**M**) of Ang II + NID or Ang II + HID mice every 7 days (n = 6; *P < 0.05, **P < 0.01 versus the Ang II + NID group). **N** and **O**: ejection fraction (EF, **N**) and fractional shortening (FS, **O**), respectively. **P** Representative images of echocardiographic images and H&E and Sirius red staining and WGA staining in the myocardium of Ang II + NID or Ang II + HID mice. (For interpretation of the references to colour in this figure legend, the reader is referred to the Web version of this article.)

In the hearts of mice receiving Ang II injections for four weeks (Ang II-induced mice, Fig. 1A), GPX4 expression was greatly decreased, HO-1 expression was increased at both the protein and mRNA levels (Fig. 1B and C), and the expression of another putative molecular marker of ferroptosis, 4-hydroxynonenal (4-HNE), was significantly enhanced in cardiac tissues, as determined by immunostaining (Fig. 1D). Additionally, iron levels in serum or heart tissues (Fig. 1E and F) and cardiac ROS levels (reflected by MDA content upregulation, SOD activity and GSH level downregulation), representing lipid peroxidation, increased in the myocardium (Fig. 1G–I). Notably, these changes were associated with increased cardiac impairment (observed by H&E staining and Sirius red staining, Fig. 1D) and decreased cardiac systolic function, as indicated by ejection fraction (EF%), fractional shortening (FS%), left ventricular internal diameter at end-diastole (LVIDd), and left ventricular end diastolic volume (LVEDV) (Figs. S1A–E).

Subsequently, we explored whether excessive iron is necessary to initiate cardiac ferroptosis. Male C57BL/6 mice were administered Ang II injections for a period of four weeks while being fed diets with varying levels of dietary iron. (Fig. 1J). As expected, compared to those fed an Ang II diet with a normal iron diet (NID), mice fed a high iron diet (HID) had no significant difference in body weight or systolic blood pressure (SBP) (Fig. 1K and L) but experienced more severe cardiac injury, as indicated by an increase in the ratio of heart weight to body weight (HW/BW) (Fig. 1M), a decrease in ventricular systolic function (based on EF%, FS%, LVIDd and LVEDV, Fig. 1N and O;Figs. S1F and G) and more severe histological damage (see H&E, Sirius Red, and WGA staining, Fig. 1P and S1H). Thus, in vivo data indicate that cardiac ferroptosis and ROS may be related to cardiac remodeling caused by Ang II.

### 3.2 Ferroptosis inhibition prevents Ang II-induced cardiac injury in mice and cultured cardiomyocytes

**Fig. 2:**
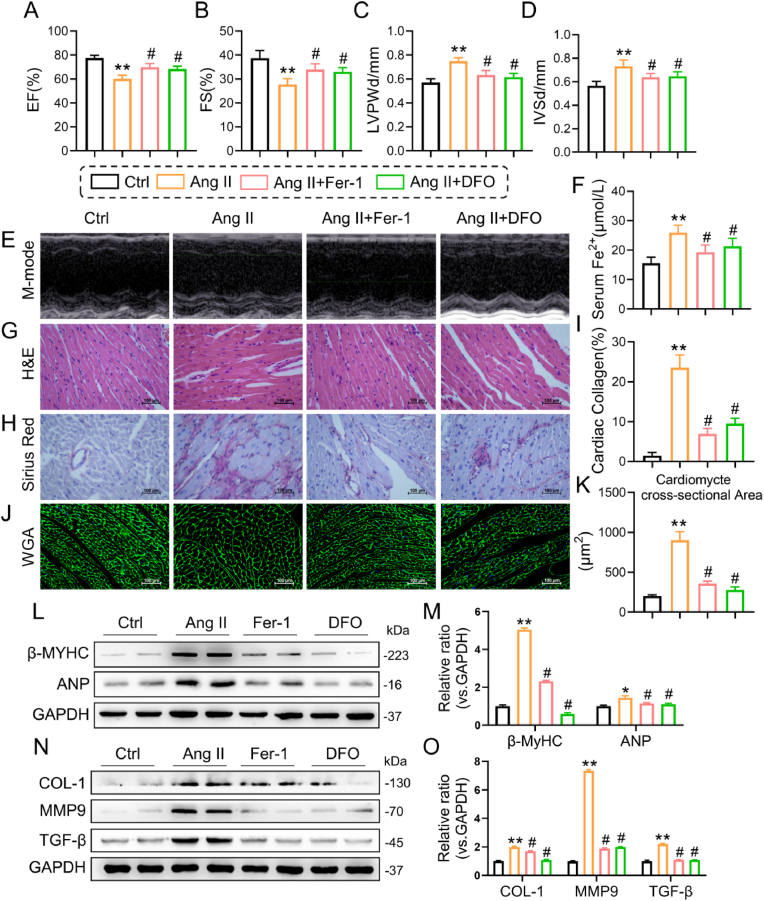
Ferroptosis inhibitors protect against Ang II-induced cardiac dysfunction and remodeling in mice. **A-D** Echocardiographic analysis of the ejection fraction (EF, **A**), fractional shortening (FS, **B**), left ventricular posterior wall thickness in diastole (LVPWd, **C**) and interventricular septal thickness at diastole (IVSd, **D**). **E** Representative M-mode echocardiographic images of each group of mice. **F** The levels of Fe^2+^ in serum. **G, I and H** Representative images of H&E and Sirius red staining and WGA in heart sections. **J** Quantification of the interstitial fibrotic area as determined by Sirius red staining. **K** Quantification of the size of myocardial cells as determined by WGA. **L** and **N** Representative Western blot analysis of β-MYHC, ANP, COL-1, MMP9 and TGF-β in cardiac tissues; GAPDH was used as a loading control. **M** and **O** Densitometric quantification of immunoblots in **L** and **N,** respectively (n = 6; *P < 0.05, **P < 0.01 versus the Ctrl group; #P < 0.05 versus the Ang II group). (For interpretation of the references to colour in this figure legend, the reader is referred to the Web version of this article.)

Ferrostatin-1 (Fer-1) and deferoxamine (DFO), ferroptosis inhibitors, were utilized to research the effects of ferroptosis on Ang II-induced cardiac remodeling. One day prior to sacrifice, we performed a noninvasive transthoracic echocardiogram on all the experimental mice to assess their heart function. Compared with Ang II injection, both Fer-1 and DFO treatment markedly improved cardiac systolic dysfunction (manifested by the EF% and FS% indices) and reduced LVPWd and IVSd (see Fig. 2A–E). Following treatment with Fer-1 or DFO, the increase in iron levels in the serum induced by Ang II was effectively reversed (Fig. 2F), accompanied by observable changes in cardiac remodeling in mice. According to histological analysis with H&E (Fig. 2G) and Sirius Red staining (Fig. 2H and I), Fer-1 or DFO treatment reversed the structural abnormalities observed in the Ang II-induced hearts, including the disorganization of myofibers (longitudinal section) and an increase in collagen deposition in the interstitial and perivascular regions of the heart. In addition, Ang II-stimulated cardiomyocyte enlargement was counteracted by Fer-1 or DFO (see the WGA results, Fig. 2J and K). In addition, profibrotic indicators, including COL-1, TGF-β, and MMP9, and prohypertrophic proteins (reflected by β-MYHC and ANP) in the heart were considerably elevated in Ang II-induced mice and dramatically reduced in Fer-1- or DFO-treated mice (Fig. 2L-O).

We pretreated primary rat cardiomyocytes and H9c2 cardiomyocytes with Fer-1 or DFO for 2 h before they were stimulated with Ang II for 24 h. The results demonstrated that Fer-1 or DFO treatment in a dose-dependent manner remarkably retarded the expression of indicators of cellular hypertrophy (β-MYHC and ANP) and matrix deposition (such as TGF-β, COL-1 and MMP9) produced by Ang II (Figs. S2A–H). Similarly, rhodamine phalloidin staining revealed that Fer-1- or DFO-pretreated H9c2 cells treated with Ang II had fewer cells (Figs. S2I and J). These in vivo and in vitro analyses revealed that mice or cardiomyocytes with ferroptosis inhibition were resistant to cardiac dysfunction and heart impairment induced by Ang II.

### 3.3 P2X7R deficiency considerably improved Ang II-induced cardiac dysfunction and cardiac remodeling in mice

**Fig. 3:**
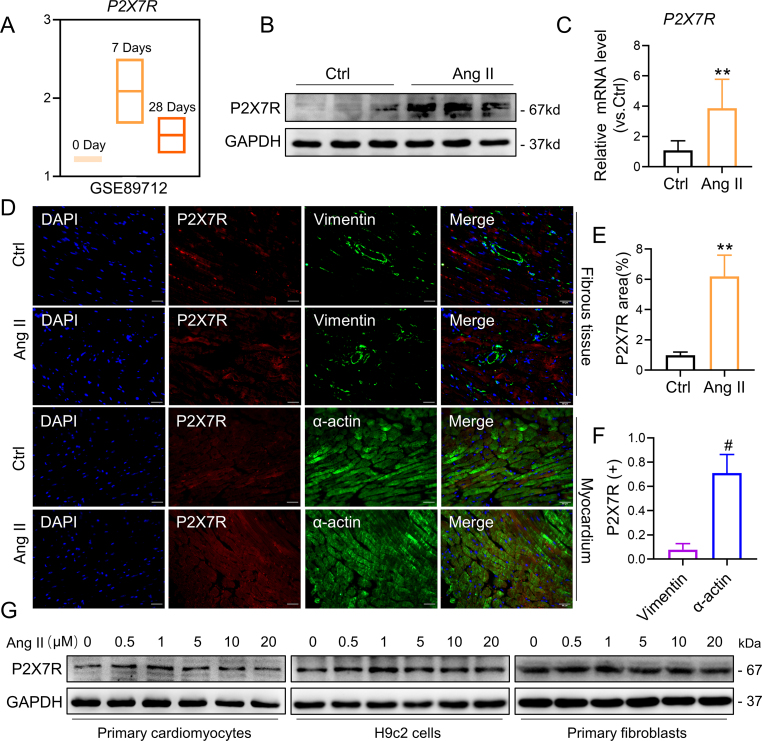
P2X7R expression was elevated in the myocardium of Ang II-infused mice and in cardiomyocytes. **A** Heatmap of P2X7R expression profiles identified in mouse models following Ang II infusion from the GSE89712 dataset (n = 2). **B** Representative immunoblot showing P2X7R in the myocardium of Ang II-infused mice. **C** mRNA levels of P2X7R in cardiac tissues. **D** Double immunofluorescence staining for P2X7R (red), the fibrosis marker vimentin (green, the top two rows) or the myocyte marker α-actin (green, the next two rows) in the myocardium of Ang II-infused mice. Merged images (orange) showing colocalization. **E** Quantification of the P2X7R immunoreactive area (%) in **D**. **F** Quantification of double immunoreactivity showing the percentages of P2X7R-positive plus α-actin- and vimentin-positive areas in images of Ang II-infused mice in **D**. **G** Representative immunoblot showing P2X7R in primary cardiomyocytes, H9c2 cardiomyocyte-like cell lines and primary fibroblasts stimulated with different concentrations of Ang II (n = 6; *P < 0.05, **P < 0.01 versus the Ctrl group; #P < 0.05 versus the Vimentin group). (For interpretation of the references to colour in this figure legend, the reader is referred to the Web version of this article.)

An analysis of RNA-seq data from three GSE89712 datasets comparing two groups of samples (ctrl and Ang II) was performed. Elevated P2X7R mRNA levels were observed in the cardiac tissue of mice that had received Ang II for four weeks (Fig. 3A). Next, we showed that the cardiac tissue of mice given Ang II had much greater levels of P2X7R protein and mRNA (Fig. 3B, C and E). Then, we used immunofluorescence to label heart tissue with either vimentin (a fibroblast marker) or α-actin (a cardiomyocyte marker) to determine the cellular origin of P2X7R in hypertrophic hearts. Most P2X7R colocalized with α-actin and exhibited high expression after Ang II infusion, confirming that cardiomyocytes are the major cell type involved in Ang II-induced P2X7R expression in cardiac tissues (Fig. 3D and F). Similar results were observed in primary cardiomyocytes and H9c2 cardiomyocytes treated with varying doses of Ang II, with increased P2X7R levels, but no changes were detected in primary fibroblasts. (Fig. 3G and Fig. S3A). Collectively, these findings suggest that Ang II elevates P2X7R expression in heart tissues, mainly in cardiomyocytes.

**Fig. 4:**
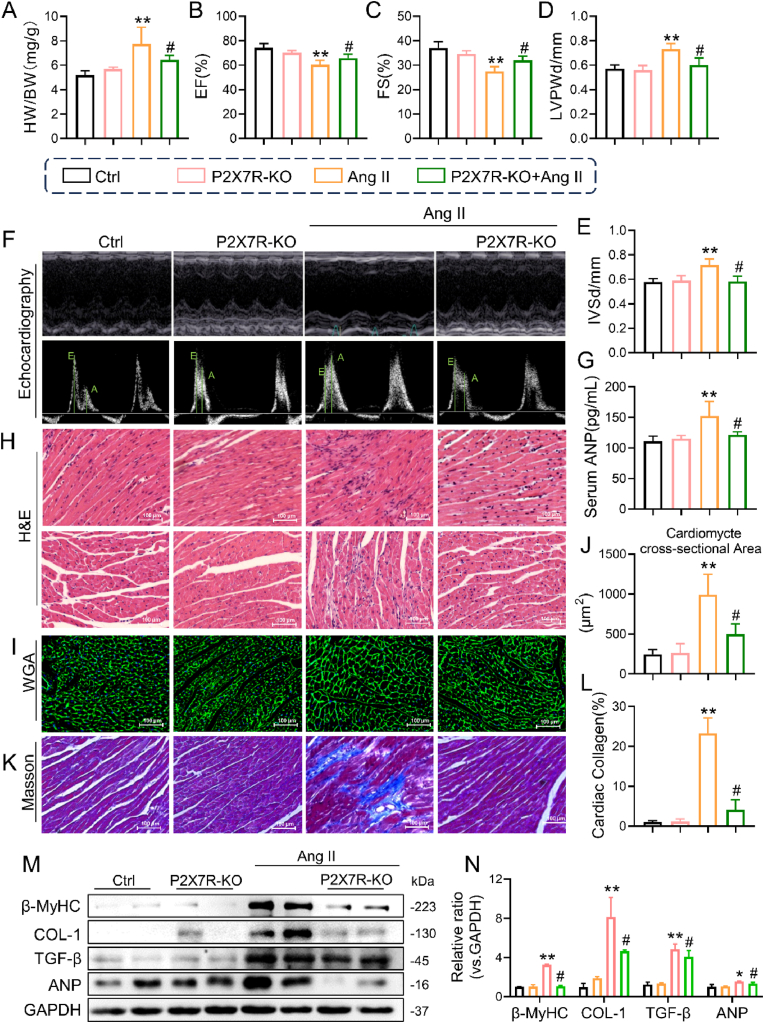
P2X7R deficiency improved Ang II-induced cardiac diastolic and systolic dysfunction and cardiac remodeling in mice. **A** C57BL/6 or P2X7R knockout mice were infused with Ang II or saline for 4 weeks. Heart weight-to-body weight ratio (HW/BW) in each group of mice. **B** Representative M-mode echocardiographic images. **C–F** Echocardiographic analysis of the ejection fraction (EF, **C**), fractional shortening (FS, **D**), left ventricular posterior wall thickness in diastole (LVPWd, **E**) and interventricular septal thickness at diastole (IVSd, **F**). **G** The serum level of ANP was detected with an ELISA kit. **H** Representative H&E-stained images (longitudinal section, upper and transverse section, bottom). **I** and **J** Representative images of WGA in transverse sections of heart tissues (**I**) and quantification of the cardiomyocyte area from WGA (**J**). **K** and **L** Representative images of Masson staining (**K**) and quantification of the interstitial fibrotic area from Masson staining (**L**). **M** Representative Western blot analysis of β-MYHC, ANP, COL-1, MMP9 and TGF-β in cardiac tissues; GAPDH was used as a loading control. **N** Densitometric quantification of immunoblots in **M** (n = 6; *P < 0.05, **P < 0.01 versus the Ctrl group; #P < 0.05 versus the Ang II group).

Using male P2X7R knockout mice (P2X7R^−/−^) and their wild-type (WT) littermates as controls (Figs. S3B–D), the importance of P2X7R in Ang II-infused mice was investigated. Both WT and P2X7R^−/−^ mice treated with Ang II exhibited increased systolic blood pressure, and both groups of mice also exhibited increased serum Ang II levels (Figs. S4A and B). Compared with that of Ang II-WT mice, the heart weight (HW)-to-body weight (BW) (HW/BW) ratio was decreased in Ang II-P2X7R^−/−^ mice, but there was no notable difference between P2X7R^−/−^ mice and WT mice (Fig. 4A). As depicted in Fig. 4B–F, Ang II injection induced diastolic dysfunction, as indicated by the LVPWd, IVSd and E/A ratio. On the other hand, Ang II injection decreased cardiac systolic function (as indicated by the EF% and FS% indices). However, both diastolic and systolic dysfunction were markedly ameliorated in P2X7R knockout mice. Additionally, we observed that the serum level of ANP, an indicator of heart injury, was increased in Ang II-induced mice but was dramatically decreased in P2X7R^−/−^ mice (Fig. 4G). Taken together, these results indicated that mice with P2X7R deficiency were protected from cardiac dysfunction and heart impairment induced by Ang II.

To examine cardiac tissue in mice, various staining techniques, including H&E staining, Masson's staining, and WGA staining, were used for histological analysis. As depicted in Fig. 4H, the hearts exposed to Ang II exhibited abnormal structures, such as irregular myofibers and increased cross-sectional areas of myofibers, which were absent in the Ang II-P2X7R^−/−^ mice. Furthermore, cross-sections subjected to H&E staining and WGA staining revealed a significant decrease in cell size in Ang II-treated P2X7R^−/−^ mice (Fig. 4H–J). In addition, the levels of ANP and β-MYHC were significantly lower in Ang II-P2X7R^−/−^ mice than in control mice (Fig. 4M and N). Masson's staining of connective heart tissues demonstrated a significant increase in collagen deposition within the cardiac interstitium of Ang II-treated mice, which was subsequently counteracted by blocking P2X7R (Fig. 4K and L). Furthermore, there was an increase in the levels of COL-1 and TGF-β in the cardiac tissues of Ang II-treated mice, but this effect was significantly diminished in mice lacking P2X7R (Fig. 4M and N). Collectively, these findings suggest that the absence of P2X7R significantly improved cardiac hypertrophy and fibrosis in Ang II-treated mice.

Additionally, we cultured primary cardiomyocytes with Ang II and observed elevated expression of COL-1, MMP9 and TGF-β, as well as of ANP and β-MYHC. Pretreatment with A438079 (a selective P2X7R inhibitor) blunted the changes in these markers in Ang II-induced cells (Figs. S4C and D). After Ang II stimulation, rhodamine phalloidin staining of A438079-pretreated H9c2 cells revealed a reduction in cell size (Figs. S4I and J). Next, we further silenced P2X7R expression in H9c2 cells via siRNA transfection (Figs. S4E and F). Silencing P2X7R markedly reduced the Ang II-induced increase in the expression of profibrotic and hypertrophic indicators, which was confirmed by decreased levels of COL-1, MMP9, TGF-β, ANP and β-MYHC (Figs. S4G and H). These results provide further evidence that P2X7R reduces Ang II-induced fibrosis and hypertrophy in cultured cardiomyocytes via both P2X7R silencing and pharmacological inhibition.

### 3.4 P2X7R deficiency greatly suppressed cardiac ferroptosis in Ang II-induced mice

**Fig. 5:**
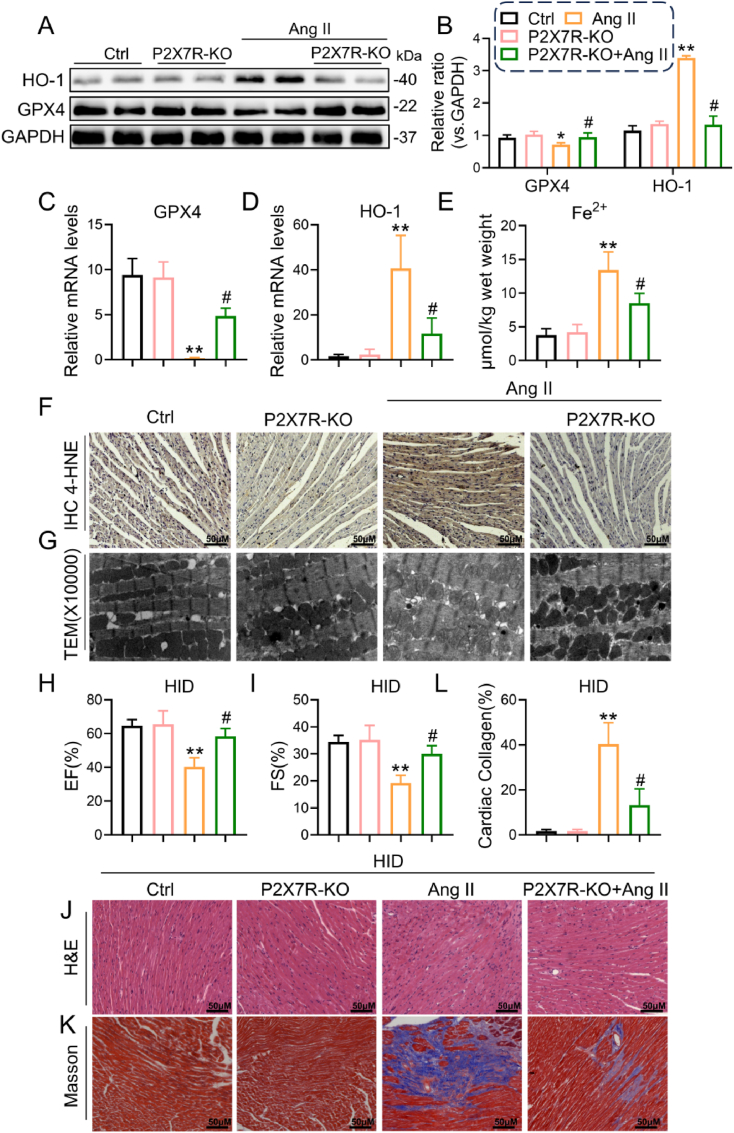
P2X7R deficiency alleviates Ang II-induced myocardial ferroptosis in Ang II-induced mice. **A** Representative Western blot analysis of HO-1 and GPX4 levels in the heart tissues of mice. GAPDH was used as a loading control. **B** Densitometric quantification of immunoblots in **A. C** and **D** mRNA levels of HO-1 and GPX4 in heart tissues. **E** The contents of Fe^2+^ in heart tissues measured by a kit. **F** Representative immunohistochemical images of 4-HNE in heart tissues. **G** Ultrastructural changes, including decreased mitochondrial volume, increased bilayer membrane density and the disappearance of mitochondrial cristae, were detected by transmission electron microscopy (TEM). **H** and **I** C57BL/6 or P2X7R knockout mice were fed a high-iron diet and infused with Ang II or saline for 4 weeks. Echocardiographic analysis of ejection fraction (EF, **H**), fractional shortening (FS, **I**) **J-L** Representative images of H&E (**J**) and Masson staining (**K**); quantification of interstitial fibrotic area from Masson staining (**L**) (n = 6; *P < 0.05, **P < 0.01 versus Ctrl group; #P < 0.05 versus Ang II group).

Our findings described above showed that Ang II led to an increase in myocardial ferroptosis, which was accompanied by elevated ROS levels and cardiac impairment, whereas both ferroptosis inhibition and P2X7R deficiency effectively improved cardiac dysfunction and heart injury caused by Ang II. Subsequently, we investigated whether overproduction of P2X7R in the myocardium participated in regulating cardiac ferroptosis. The cardiac levels of GPX4, HO-1 and 4-HNE were determined by Western blotting, RT‒PCR and immunohistochemical staining, respectively. As expected, a decrease in GPX4 and an increase in HO-1 and 4-HNE in cardiac tissues with Ang II stimulation were abolished in Ang II-P2X7R^−/−^ mice; however, P2X7R deficiency without Ang II treatment did not affect the levels of these proteins (similar to those in the control group) in the hearts of mice (Fig. 5A–D and Fig. 5F). Additionally, the infusion of Ang II led to an increase in Fe^2+^ levels in cardiac tissues, which was subsequently reversed by P2X7R deficiency (Fig. 5E). Next, we examined the ultrastructure of myocardial mitochondria through TEM analysis, and the findings revealed that heart sections from the Ang II group exhibited a decrease in mitochondrial volume, an increase in bilayer membrane density, and a reduction or absence of mitochondrial cristae (Fig. 5G). However, in the myocardium of Ang II-P2X7R^−/−^ mice, ultrastructural alterations in mitochondrial organization were obviously improved. These data suggested that P2X7R blockade can inhibit cardiac ferroptosis induced by Ang II in mice.

Furthermore, according to our above results that mice with Ang II which were given a HID contributed to severer cardiac injury, we also gave male C57BL/6 or P2X7R knockout mice a high-iron diet and then infused these mice with Ang II or saline. As depicted in Fig. 5H-L, compared to HID (as a control) mice, Ang II + HID-treated mice exhibited obvious decreases in EF% and FS%, accompanied by an increase in cardiac fibrosis, while P2X7R knockout strongly reversed Ang II + HID-induced cardiac systolic dysfunction and was accompanied by the remission of cardiac fibrosis, suggesting that P2X7R deficiency alleviated the Ang II-induced worsening of heart damage in iron-loaded diet-fed mice.

**Fig. 6:**
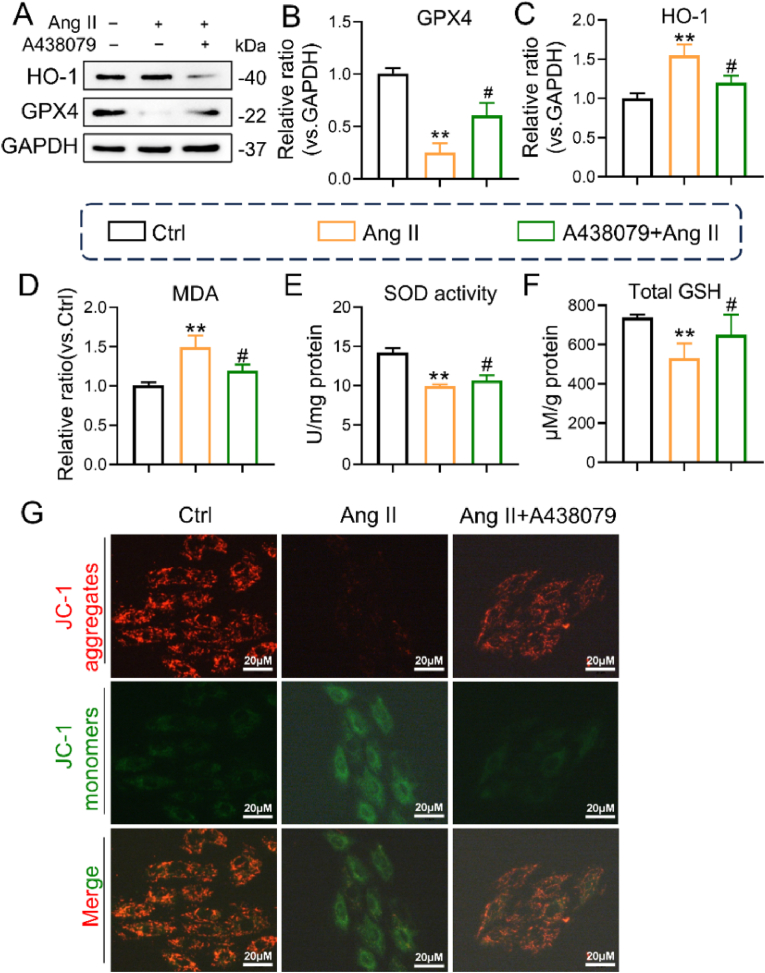
P2X7R inhibition abolished Ang II-induced ferroptosis in vitro. H9c2 cells were pretreated with 10 μM A438079 (a P2X7R inhibitor) for 2 h and then stimulated with 1 μM Ang II for 24 h. **A** Representative Western blot analysis of HO-1 and GPX4 levels; GAPDH was used as a loading control. **B** and **C** Densitometric quantification of immunoblots in **A**. **D-F** Representative MDA content, SOD activity and GSH levels in each group. **G** The mitochondrial membrane potential of H9c2 cells was measured by JC-1 staining (n = 3; *P < 0.05, **P < 0.01 versus the Ctrl group; #P < 0.05 versus the Ang II group).

We pretreated cultured H9c2 cells with A438079 for pharmacological inhibition, followed by stimulation with Ang II for 24 h, and we observed similar results for GPX4 and HO-1 protein levels in vivo (Fig. 6A–C). Next, we also found that SOD activity and GSH levels were significantly reduced and that MDA was increased, and these alterations were blunted by A438079 in Ang II-stimulated cells (Fig. 6D–F). Additionally, we observed a reduction in the mitochondrial membrane potential in H9c2 cells following Ang II stimulation, which was subsequently restored by A438079 (Fig. 6G). Thus, these data from both mice and cells unequivocally demonstrate that P2X7R plays a crucial role in Ang II-induced cardiac ferroptosis by normalizing GPX4 and HO-1 and balancing ROS levels.

### 3.5 P2X7R blockade decreased GPX4 mRNA degradation but increased HO-1 mRNA degradation in Ang II-induced cardiomyocytes by affecting HuR

**Fig. 7:**
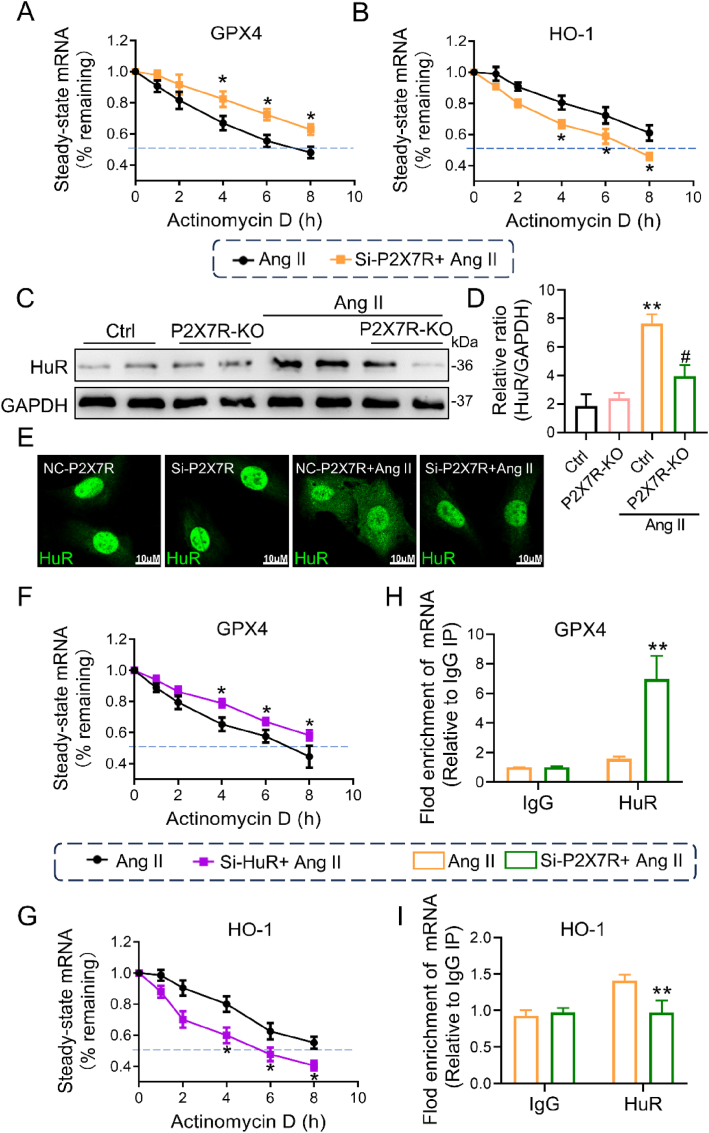
P2X7R blockade affects the stability of GPX4 and HO-1 mRNA by regulating HuR expression and nucleocytoplasmic shuttling. **A** and **B** H9c2 cells transfected with si-P2X7R were stimulated with 1 μM Ang Ⅱ for 24 h, and actinomycin D (Act D) was added at different times to interfere with the cell transcriptional cycle. The mRNA levels of GPX4 (**A**) and HO-1 (**B**) in H9c2 cells were detected. **C** Representative Western blot analysis of HuR levels with GAPDH as a loading control. **D** Densitometric quantification of immunoblots in **C**. **E** Immunofluorescence staining for HuR (green) in H9c2 cells. Increased fluorescence intensity in the cytoplasm after stimulation with 1 μM Ang Ⅱ. **F** and **G,** Relative GPX4 mRNA (**F**) and HO-1 mRNA (**G**) in H9c2 cells transfected with Si-HuR, stimulated with Ang Ⅱ and then treated with Act D. **H** and **I** RNA binding protein immunoprecipitation assays were used to verify the binding of HuR to GPX4 (**H**) and HO-1 (**I**) mRNA (n = 3; *P < 0.05, **P < 0.01 versus the Ctrl group; #P < 0.05 versus the Ang II group). (For interpretation of the references to colour in this figure legend, the reader is referred to the Web version of this article.)

There is a pressing need to understand how P2X7R blockade inhibits ferroptosis. Based on our previous data, knockdown of P2X7R resulted in changes in both the protein and mRNA levels of GPX4 and HO-1, which are recognized as crucial markers associated with ferroptosis. Consequently, we sought to investigate whether P2X7R could modulate the transcriptional regulation of GPX4 and HO-1, thereby influencing their mRNA and protein expression. Therefore, we further investigated the impact of P2X7R on the stability of HO-1 and GPX4 mRNA. To this end, we transfected H9c2 cells with the appropriate P2X7R-siRNA and stimulated them with Ang II, after which the cells were exposed to actinomycin D. The half-lives of GPX4 and HO-1 mRNA were then determined by collecting total cellular RNA at the appropriate times and using real-time PCR. As depicted in Fig. 7A and B, in cardiomyocytes from the Ang II + siP2X7R group, the half-life of GPX4 mRNA was significantly greater than that in cardiomyocytes from the Ang II group, but the half-life of HO-1 mRNA was shorter, indicating that P2X7R silencing increased the stability of GPX4 mRNA and decreased the stability of HO-1 mRNA.

Given that mRNA binding sites and AU-rich elements (AREs) in the 3′-UTR of certain mRNAs are common genetic factors influencing mRNA stability, we identified human antigen R (HuR), a relatively common AU-rich element-binding protein (AUBP), as a target for subsequent investigations. HuR, a protein that binds to mRNA, is a crucial regulator of several pathophysiological conditions through the binding of U- and AU-rich elements in its 3′-untranslated region (UTR), which alters relative target mRNA stability or degradation [23,24]. Therefore, we investigated whether P2X7R blockade results in a change in HuR expression in cardiac tissues and influences the shuttling of cytoplasmic HuR. As shown in Fig. 7C and D, Ang II-treated mouse hearts exhibited noticeably increased levels of HuR expression, which was restored in Ang II-P2X7R knockout mice. Additionally, Ang II enhanced HuR levels in H9c2 cells and triggered a distinct increase in the transfer of HuR from the nucleus to the cytoplasm, which was markedly abrogated by siP2X7R (Fig. 7E). These observations suggested that P2X7R deficiency effectively regulated cytoplasmic HuR accumulation and expression. In addition, we found that the effective reduction of HuR using HuR siRNA also increased the decrease in HO-1 mRNA and reduced GPX4 mRNA degradation in Ang II-treated H9c2 cells (Fig. 7F and G). Furthermore, independent assessment of the binding of HuR to GPX4 and HO-1 mRNA was performed through a RIP assay. As shown in Fig. 7H and I, HuR can directly interact with GPX4 and HO-1 mRNA. With the use of P2X7R siRNA, the interaction between HuR and GPX4 mRNA increased, whereas the interaction between HuR and HO-1 mRNA decreased. These results supported the view that P2X7R blockade regulated the stability of GPX4 and HO-1 mRNA by altering the binding of HuR to the corresponding mRNAs.

### 3.6 Ang II directly interacts with P2X7R at positions LYS-66 and MET-212

**Fig. 8:**
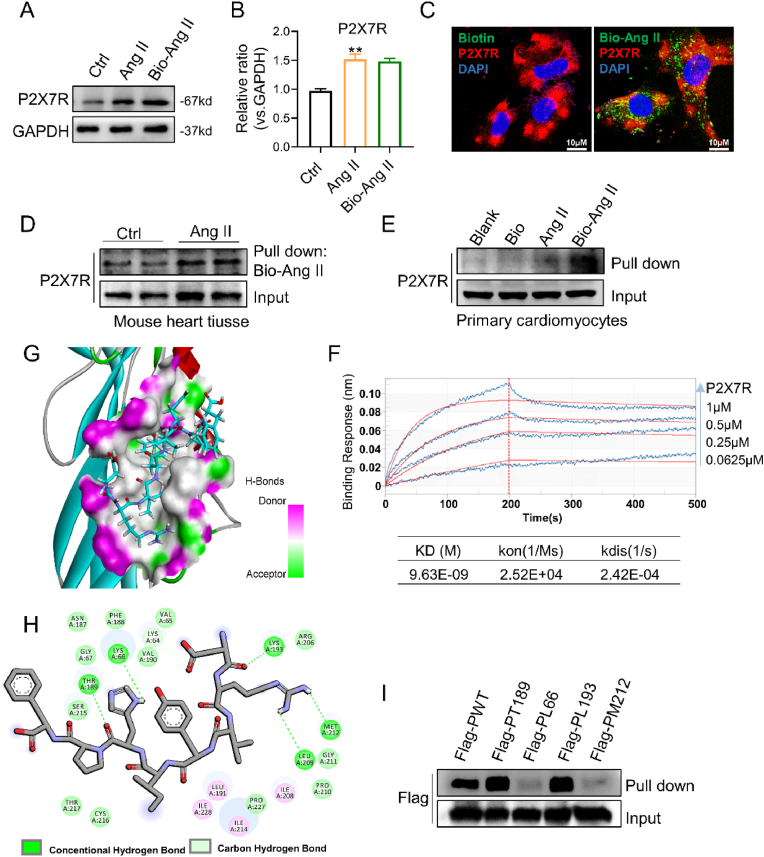
Ang II directly binds to the P2X7R protein at positions LYS-66 and MET-212. **A** and **B** H9c2 cells were treated with 1 μM Ang II or biotinylated angiotensin II (Bio-Ang II) for 24 h. Representative Western blot analysis of P2X7R in cells with GAPDH as a loading control (**A**). Densitometric quantification of immunoblots in **A** (**B**) (n = 3; **P < 0.01 versus the Ctrl group). **C** H9c2 cells were treated with 1 μM Bio-Ang II or free biotin for 24 h, followed by double-immunofluorescence staining for biotin (green) and P2X7R (red). **D** and **F** Binding of Bio-Ang II to P2X7R was determined by pull-down assays. Bio-Ang II was added to streptavidin-agarose beads, and total lysates were used as an input control. Lysates prepared from heart tissues of mice infused with saline or Ang II were added to streptavidin-agarose beads with Bio-Ang II (**D**). Lysates were prepared from primary cardiomyocytes overexpressing Flag-P2X7R. Untreated beads (Blank), biotin alone (Bio) and unconjugated Ang II (Ang II) were used as controls (**E**). **F** Biolayer interferometry (BLI) analysis of the binding of Ang II to purified P2X7R protein. Ang II was added at different concentrations, and kinetic analysis was performed (shown in the panel below). **G** and **H** Molecular docking study between compound Ang II and the 3D structure of P2RX7 (PDB code: 6U9W) (**G**). The key amino acids are connected to the molecular formula of Ang II through dashed lines (**H**). **I** HEK-293T cells were transfected with Flag-tagged P2X7R with THR-189 (Flag-PT189), Flag-tagged P2X7R with LYS-66 (Flag-PL66), Flag-tagged P2X7R with LYS-193 (Flag-PL193) and Flag-tagged P2RX7 with MET-212 (Flag-PM212). Lysates were added to streptavidin-agarose beads with Bio-Ang II. **H** Schematic representation of the key findings of this study. Ang II-induced myocardial remodeling involves upregulation of P2X7R expression and activation of ferroptosis. Intriguingly, Ang II directly interacts with the P2X7R protein. Furthermore, P2X7R mediates Ang II-induced myocardial ferroptosis through HuR, which affects the stability of GPX4 and HO-1 mRNA. (For interpretation of the references to colour in this figure legend, the reader is referred to the Web version of this article.)

Then, we used a biotinylated form of Ang II (Bio-Ang II) to examine the molecular connection between Ang II and P2X7R. Additionally, enhanced P2X7R expression in myocytes exposed to Bio-Ang II maintains the effect of Ang II. (Fig. 8A and B). After H9c2 cells were treated with biotin or Bio-Ang II, the cells were subjected to immunofluorescence staining for P2X7R and Bio-Ang II. These proteins may colocalize on the cell surface, suggesting that Ang II and P2X7R may interact (Fig. 8C). To determine whether Ang II binds to P2X7R, we also conducted pull-down assays with protein lysates obtained from primary cardiomyocytes and the myocardium of mice treated with Ang II. The results demonstrated that in these lysates, Bio-Ang II bound to the P2X7R protein (Fig. 8D and E). These findings demonstrate that Ang II directly interacts with P2X7R to promote its expression.

Furthermore, we employed biolayer interferometry (BLI) to examine the nature of the binding between Ang II and P2X7R. BLI analysis revealed that the purified P2X7R protein (extracellular domain) was directly bound by Ang II, with a KD value of 9.63 nM (Fig. 8F). Next, to further strengthen this interaction, we conducted a molecular docking study between compound Ang II and the 3D structure of P2X7R (PDB code: 6U9 W). As shown in Fig. 8G and H, the docking scores indicate that Ang II could connect with the extracellular domain of P2X7R and be well embedded in the ATP binding pocket of P2X7R through five hydrogen bonds with LYS-66, THR-189, LYS-193, LEU-209 and MET-212, and the importance of five key residues in the extracellular domains of P2X7R was identified as THR-189 > LYS-66 > LYS-193 > MET-212 > LEU-209 according to the predicted average energy values. Among the five key residues, we selected THR-189, LYS-66, LYS-193 and MET-212 according to their predicted average energy values and then individually mutated THR-189, LYS-66, LYS-193 and MET-212 to alanine (ALA) to investigate their roles in the Ang II/P2X7R interaction.

Subsequently, we constructed four independent mutant plasmids, Flag-tagged P2X7R with THR-189 (Flag-PT189), Flag-tagged P2X7R with LYS-66 (Flag-PL66), Flag-tagged P2X7R with LYS-193 (Flag-PL193) and Flag-tagged P2X7R with MET-212 (Flag-PM212), and transfected them into HEK-293T cells. Interestingly, we discovered that LYS-66 or MET-212 mutations, but not THR-189 or MET-212 mutations, significantly reduced the interaction between Ang II and P2X7R (Fig. 8I), suggesting that LYS-66 or MET-212 is primarily involved in Ang II binding. In summary, these results offer compelling evidence of the direct effects of Ang II and P2X7R.

## 4 Discussion

This study showed that P2X7R and myocardial ferroptosis are crucial for cardiac remodeling induced by Ang II. P2X7R deficiency significantly reduced cardiac ferroptosis in mice and cardiomyocytes. Importantly, Ang II activated the transport of HuR from the nucleus to the cytoplasm and enhanced its expression, thereby increasing HO-1 mRNA and decreasing GPX4 mRNA levels. The inhibition or deletion of P2X7R decreased the amount of Ang II-induced HuR expression and regulated the binding of HuR to GPX4 and HO-1 mRNA, which then restored the expression of GPX4 and HO-1, ultimately leading to the alleviation of phenotypes, such as hypertrophy and fibrosis, and improved cardiac dysfunction. Notably, Ang II directly interacts with the extracellular domain of P2X7R to promote HuR nucleocytoplasmic shuttling in myocytes, and we identified LYS-66 or MET-212 on P2X7R as critical residues contributing to Ang II binding.

Ang II can directly trigger cardiac remodeling independently of its hemodynamic effects, thus propagating extracellular matrix accumulation either by promoting collagen expression or via the induction of plasminogen activator inhibitors [25] or myeloid differentiation 2 (MD2) [26]. Notably, P2X7R is a distinct P2X family member that can be found both inside cells and in the plasma membrane [27,28] and contributes to kidney damage caused by high sugar levels or hypertension, and P2X7R blockade inhibits renal macrophage accumulation and results in a reduction in renal injury in patients with diabetes [29,30]. Numerous chronic inflammatory diseases, such as Alzheimer's disease, multiple sclerosis, and arthritis, have been linked to the progression of P2X7R activation [31]. In addition, emerging evidence has shown that P2X7R activation negatively impacts the cardiovascular system, for instance, causing autoimmunity in people with dilated cardiomyopathy [32] and contributing to atherosclerotic plaque formation and rupture by mediating inflammation and endothelial dysfunction [[33], [34], [35]]. In addition, P2X7R activation enhances cardiomyocyte apoptosis, leading to cardiac dysfunction in acute myocardial infarction [18,35,36]. Therefore, directed P2X7R inhibition may represent an appropriate target for treating various cardiovascular disorders. In the present study, we found that Ang II administration greatly increased P2X7R at the protein and mRNA levels, which occurred simultaneously with the progression of cardiac remodeling and dysfunction in mice, mainly in cardiomyocytes (not in fibroblasts), and P2X7R knockout or pharmacological inhibition (A438079) obviously reversed the reduction in cardiac remodeling and dysfunction induced by Ang II. Additionally, the knockdown of P2X7R did not affect blood pressure or serum Ang II levels in Ang II-infused mice, suggesting that the improvement in Ang II-induced ventricular remodeling induced by P2X7R knockout is unaffected by the hemodynamic effects of Ang II. Furthermore, we identified a direct connection between Ang II and P2X7R proteins via a sequence of biochemical tests, indicating that Ang II serves as a novel activating ligand for P2X7R in cardiomyocytes. Our results showed that the key residues LYS-66 and MET-212 are involved in the interaction between Ang II and P2X7R. Thus, P2X7R acts as a new myocardial binding site for Ang II to promote cardiac remodeling. Therefore, this discovery may help in the design of inhibitors that act against P2X7R or interfere with the binding of Ang II to P2X7R, which can improve cardiac dysfunction.

To further understand how P2X7R contributes to cardiac fibrosis and hypertrophy, ferroptosis in cardiomyocytes was examined. Ferroptosis is a newly discovered form of controlled cell death that is characterized by iron overload and occurs in the presence of high levels of lipid peroxide [9,37]. A high-iron diet exacerbates angiotensin II- or DOX-induced cardiac injury, and ferritin H deficiency facilitates hypertrophic cardiomyopathy; subsequently, these aberrant phenotypes are rescued by Fer-1 [14,37,38]. Furthermore, Zhang et al. showed that Ang II enhanced ferroptosis in cardiac microvascular endothelial cells and that this effect was alleviated by elabela [15]. In our current investigation, we also discovered that cardiac Fe^2+^ content, HO-1, and 4-HNE were significantly increased, and lipid peroxides were upregulated; in contrast, GPX4 expression was decreased in the Ang II-infused model, and a comparable pattern was found in cardiomyocytes. Additionally, a high-iron diet further augmented cardiac fibrosis and dysfunction caused by Ang II treatment, and ferroptosis inhibitors (Fer-1 or DFO) significantly reversed these aberrant alterations. Taken together, these findings suggest that Ang II stimulation promotes cardiac ferroptosis and that inhibition of ferroptosis is beneficial for improving cardiac remodeling. Importantly, similar to Fer-1 and DFO, P2X7R knockout in mice or P2X7R inhibition significantly suppressed cardiac Fe^2+^ content and HO-1 and 4-HNE levels and upregulated GPX4 expression. Moreover, a reduction in ROS (as evidenced by GSH and SOD upregulation and MDA inhibition) improved abnormal mitochondria, suggesting that cardiac P2X7R, an upstream regulator, positively mediates cardiac ferroptosis in Ang II-induced mice. In addition, the heart is a very well-organized organ with various cells, consisting mainly of myocytes, endothelial cells, fibroblasts, and inflammatory cells [39]. Zhong et al. showed that elabela plays an antifibrotic role in hypertensive mice, mainly through its negative regulation of ferroptosis in cardiac microvascular endothelial cells [15]. In contrast, we found that the disruption of cardiac ferroptosis was mainly present in myocytes because P2X7R expression was obviously increased in the myocytes of mice and in cultured H9c2 cells stimulated with Ang II but not in fibroblasts. In line with findings in mice, a P2X7R inhibitor (A438079) reduced ferroptosis and exhibited antifibrotic and antihypertrophic effects on myocytes induced by Ang II. These data provide evidence that P2X7R deficiency inhibits myocardial ferroptosis and improves cardiac remodeling and dysfunction. However, the P2X7R–KO mice in our study were global rather than cardiomyocyte specific, which is one of the limitations of this study.

However, the mechanism by which P2X7R regulates cardiac ferroptosis in an Ang II-induced model remains unclear. The RNA binding protein HuR, which is broadly distributed, binds to particular AU-rich domains in a variety of mRNAs. Under basal conditions, HuR is mostly concentrated in the nucleus, and nucleocytoplasmic shuttling is a key prerequisite for regulating mRNA stability and/or translation [23,40,41]. For instance, HuR increases the expression of numerous apoptosis-inhibiting proteins but inhibits the expression of several apoptosis-promoting proteins [42], indicating that HuR plays a dual modulatory role by altering the quantity of HuR-mRNA complexes in the cytoplasm. Earlier investigations have shown that HuR functions as a central regulator of cardiac pathology. Krishnamurthy et al. indicated that HuR enhancement in cardiomyocytes after ischemia/reperfusion injury contributes functionally to promoting postinfarct remodeling [43,44]. Additionally, a strong positive correlation has been observed between HuR expression and the progression of pathological cardiac hypertrophy [45,46]. In this study, we found that Ang II caused a considerable increase in HuR expression in heart tissue and enhanced the nucleocytoplasmic shuttling of HuR in cardiomyocytes, and these effects were reversed by P2X7R deletion or pharmacological inhibition, suggesting that the P2X7R-mediated regulation of ferroptosis and cardiac remodeling may depend heavily on cardiac HuR. Interestingly, both P2X7R deficiency and HuR silencing in cardiomyocytes effectively decreased the quantity of HO-1; in contrast, the expression of GPX4 increased. Given that the 3’ UTRs of the mRNAs of these genes are rich in U or AU elements that interact with HuR, we postulated that the impact of P2X7R on Ang II-induced cardiac remodeling and myocardial ferroptosis is related to the binding of HuR to the mRNA and influences the relative stability of the target mRNAs. As expected, we demonstrated that siRNA-HuR transfection decreased the stability of HO-1 mRNA and reduced GPX4 mRNA decay in Ang II-induced myocytes, eventually leading to changes in protein levels. In addition, the RIP results showed that P2X7R suppression led to a decrease in the binding of HuR to HO-1 mRNA and an increase in the binding of HuR to GPX4 mRNA, which resulted in changes in HO-1 and GPX4 levels. Thus, HuR binding destabilized GPX4 mRNA and stabilized HO-1 mRNA in Ang II-triggered myocytes. Here, we first provided evidence for the relevance of HuR in the posttranscriptional regulation of HO-1 and GPX4 expression in an Ang II-stimulated model and demonstrated that HuR can bidirectionally regulate mRNA stability. These findings strongly indicate that P2X7R regulates cardiac remodeling and ferroptosis by influencing the capacity of HuR to bind to its target mRNA. Additionally, we may further explore the connection between P2X7R and the regulation of HuR expression in our future investigations, and HuR is likely to be a new significant target for Ang II-induced cardiac remodeling.

In summary, to our knowledge, our findings establish for the first time that P2X7R, a novel nonclassical receptor of Ang II, promotes cardiac hypertrophy and fibrosis and that its detrimental effects are induced by an increase in myocardial ferroptosis. In addition, we demonstrated that HuR is an mRNA stabilizing regulator that influences the stability of HO-1 and GPX4 mRNAs through posttranscriptional regulation, thereby leading to cardiac ferroptosis, hypertrophic responses and mitochondrial dysfunction. In our study, we provide (1) strong evidence of the pivotal role of P2X7R in Ang II-induced cardiac ferroptosis and remodeling through direct Ang II binding, (2) an in-depth description of how Ang II enhances myocardial ferroptosis and thus contributes to cardiac dysfunction, and (3) evidence that P2X7R could become a promising novel target for managing hypertensive cardiac injury.

## Sources of Funding

This work was supported by the 10.13039/501100004731Natural Science Foundation of Zhejiang Province [Grant No. LY22H020004 and LGF22H020016], the 10.13039/501100001809National Natural Science Foundation of China [Grant No. 82070446 and 82202380], the Traditional Chinese Medicine Administration of Zhejiang Province [Grant No. 2022ZA096], and the Science and Technology Project of Wenzhou [Grant No. Y20210136].

## CRediT authorship contribution statement

**Xin Zhong:** Writing – original draft, Investigation. **Kangwei Wang:** Software, Investigation. **Yonghua Wang:** Supervision. **Luya Wang:** Methodology. **Sudan Wang:** Formal analysis. **Weijian Huang:** Supervision, Funding acquisition. **Zhuyin Jia:** Supervision, Funding acquisition. **Shan-Shan Dai:** Resources, Funding acquisition. **Zhouqing Huang:** Writing – review & editing, Funding acquisition.

## Declaration of competing interest

The study's authors state that there were no financial or commercial ties that would raise the possibility of a conflict of interest throughout its execution. Regarding this paper, the authors disclose no conflicts of interest.

## Data Availability

The data that has been used is confidential.
