# Supplementary material for: Angiotension II directly bind P2X7 receptor to induce myocardial ferroptosis and remodeling by activating human antigen R
Source: Redox Biol. 2024 Apr 9;72:103154. doi: 10.1016/j.redox.2024.103154 (PMC11035111; doi:10.1016/j.redox.2024.103154)

**Table S1 Sequences of primers utilized for qPCR in the study**

| **Gene** | **Species** | **Sequence** |
| --- | --- | --- |
| *Gpx4* | Rat | CCGGCTACAATGTCAGGTTT  ACGCAGCCGTTGTTATCAAT |
| *Gpx4* | Mouse | GATGGAGCCCATTCCTGAACC  CCCTGTACTTATCCAGGCAGA |
| *Hmox1* | Rat | AGGTGCACATCCGTGCAGAG  CTTCCAGGGCCGTATAGATATGGTA |
| *Hmox1* | Mouse | GGTGATGGCTTCCTTGTACC  AGTGAGGCCCATACCAGAAG |
| *Gapdh* | Rat | CCGCATCTTCTTGTGCAGTG  GAGAAGGCAGCCCTGGTAAC |
| *Gapdh* | Mouse | AGGTCGGTGTGAACGGATTTG  TGTAGACCATGTAGTTGAGGTCA |

**Figure S1 Iron supplementation aggravates cardiac dysfunction in Ang II-induced Mice**

**A** Representative M-mode echocardiographic images of Ctrl or Ang II mice. **B-D** Echocardiographic analysis of the ejection fraction (EF, **B**), fractional shortening (FS, **C**), left ventricular internal diameter at end-diastole (LVIDd, **D**) and left ventricular end diastolic volume (LVEDV, **E**) of Ctrl or Ang II mice (n=6; *versus the Ctrl group; *P<0.05, **P<0.01). **F** and **G** Echocardiographic analysis of the LVIDd (**F**) and LVEDV (**G**) of Ang II+NID or Ang II+HID mice. **H** Quantification of the cardiomyocyte area from WGA (n=6; *P<0.05, **P<0.01 versus the Ang II+NID group).

**Figure S2 Ferroptosis inhibitors protect against Ang II-induced cardiac hypertrophy in cells.**

**A-D** Primary rat cardiomyocytes were pretreated with Fer-1 or DFO for 1 h and then cultured with 1 µM Ang II for 24 h. Representative Western blot analysis of β-MyHC, ANP, COL-1, MMP9 and TGF-β in cells with GAPDH as a loading control (**A**, **C**); densitometric quantification of immunoblots in **A** and **C** (**B**, **D**). **E**-**H** H9c2 cells were pretreated with Fer-1 or DFO for 1 h and then cultured with 1 µM Ang II for 24 h. Representative Western blot analysis of β-MyHC, ANP, COL-1, MMP9 and TGF-β in cells with GAPDH as a loading control (**E**, **G**); densitometric quantification of immunoblots in **E** and **G** (**F**, **H**). **I** and **J** Rhodamine phalloidin staining of H9c2 cells in the indicated groups (**I**); quantification of the cell area after rhodamine phalloidin staining (**J**) (n=3; *P<0.05, **P<0.01 versus the Ctrl group; #P<0.05 versus the Ang II group).

**Figure S3 P2X7R protein levels in cardiac tissue and various heart cells stimulated with Ang II.**

**A** Densitometric quantification of the immunoblots shown in **Figure 3G** (n=3; *P<0.05, **P<0.01 versus the Ctrl group; #P<0.05 versus the Ang II group). **B** Images of gene identification from male P2X7R knockout mice (P2X7R^-/-^) and their control wild-type (WT) littermates. **C** Representative Western blot analysis of P2X7R in heart tissues from WT and P2X7R^-/-^ mice; GAPDH was used as a loading control. **D** Densitometric quantification of immunoblots in **C**.

**Figure S4 P2X7R inhibition alleviates Ang II-induced cardiac hypertrophy in cells.**

**A** Systolic blood pressure was measured weekly by a noninvasive tail-cuff pressure analysis system. **B** The serum Ang II level in the mice was determined with an ELISA kit (n=6; *versus the Ctrl group; *P<0.05 and **P<0.01). **C** Primary rat cardiomyocytes were pretreated with 10 µM A438079 for 1 h and stimulated with 1 µM Ang II for 24 h. Representative Western blot analysis of β-MyHC, ANP, COL-1, MMP9 and TGF-β in cells with GAPDH as a loading control. **D** Densitometric quantification of immunoblots in **C**. **E** P2X7R was knocked down in H9c2 cells by P2X7R siRNA-1, siRNA-2 or siRNA-3 (si-P2X7R-1, -2, and -3, respectively). Ctrl cells were left untreated. NC cells were transfected with negative control siRNA. Representative Western blot analysis of P2X7R in cells with GAPDH as a loading control. **F** Densitometric quantification of immunoblots in **E**. **G** H9c2 cells transfected with si-P2X7R were stimulated with 1 µM Ang Ⅱ for 24 hours. Representative Western blot analysis of β-MyHC, ANP, COL-1, MMP9 and TGF-β in cells with GAPDH as a loading control. **H** Densitometric quantification of immunoblots in **G**. **I** and **J** Rhodamine phalloidin staining of H9c2 cells in the indicated groups (**I**); Quantification of the cell area by rhodamine phalloidin staining (**J**) (n=3; *P<0.05, **P<0.01 versus the Ctrl group; #P<0.05 versus the Ang II group).

**Figure S1**


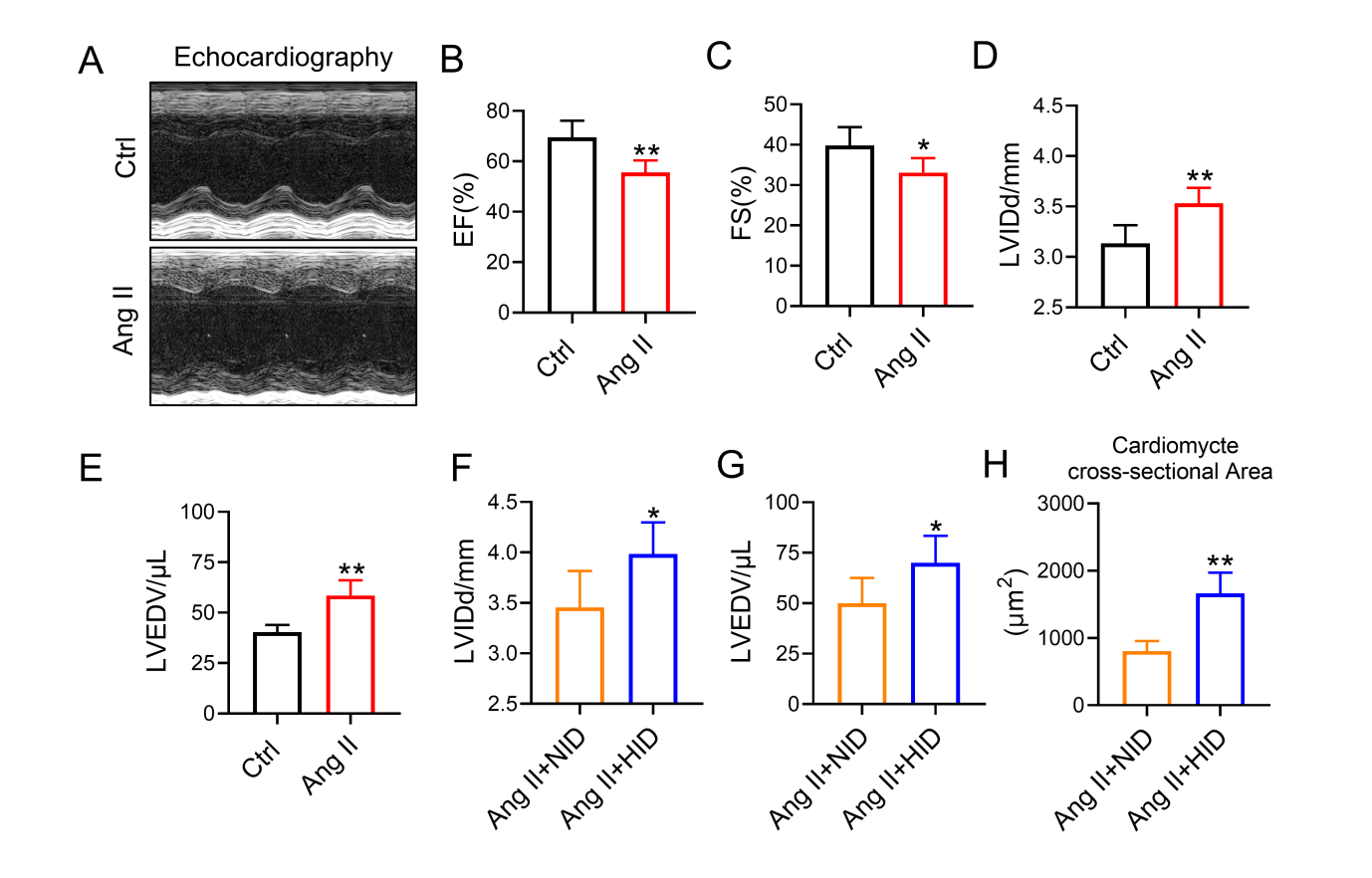


**Figure S2**


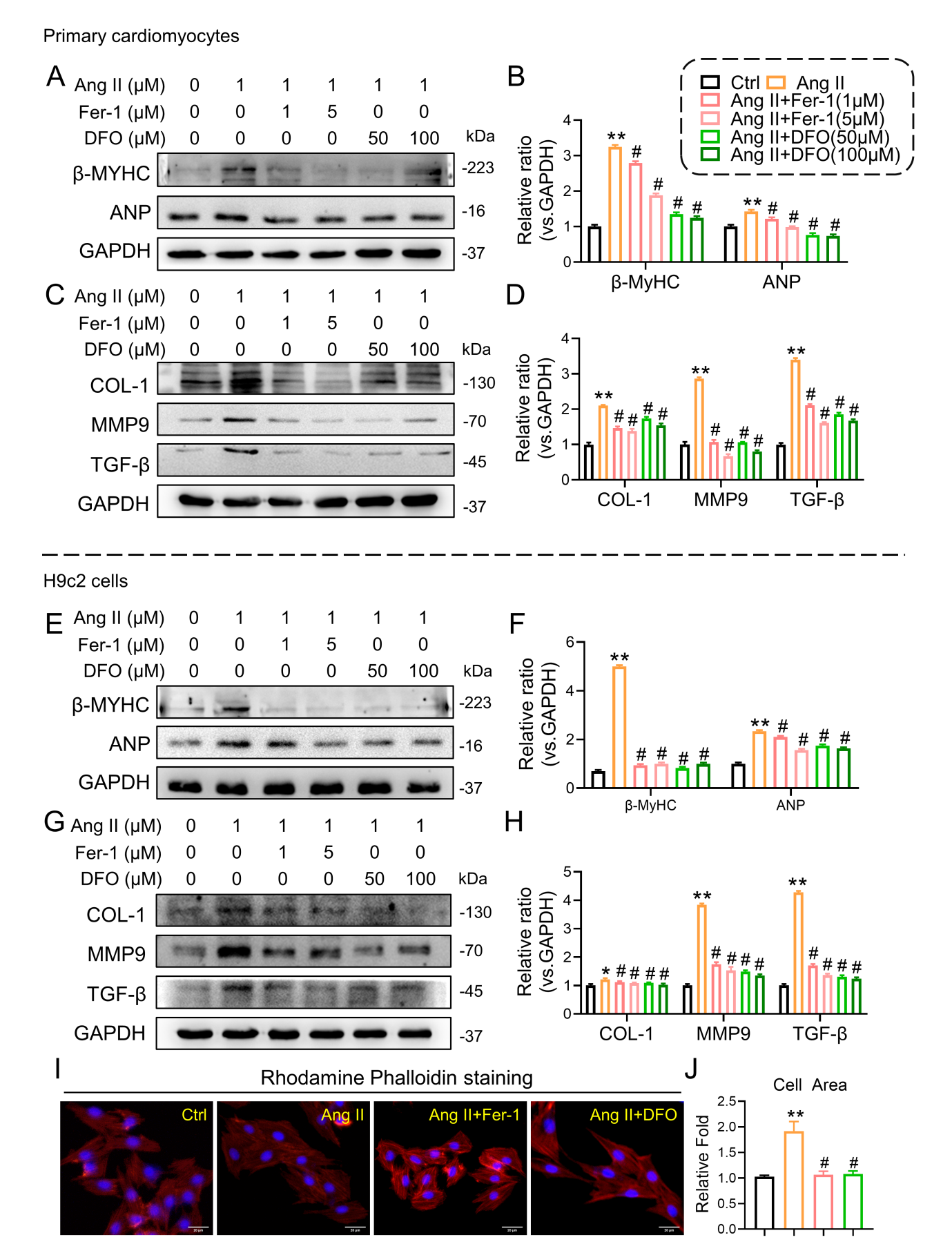


**Figure S3**


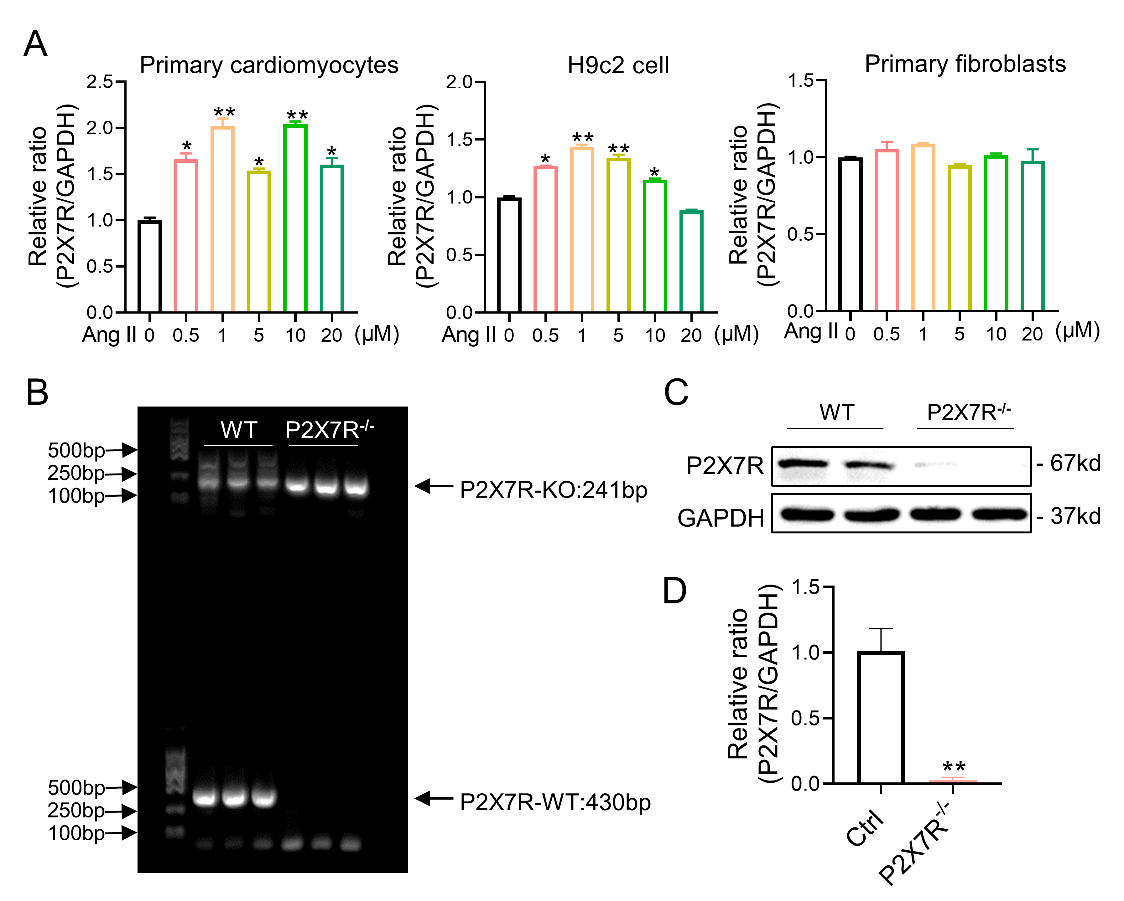


**Figure S4**


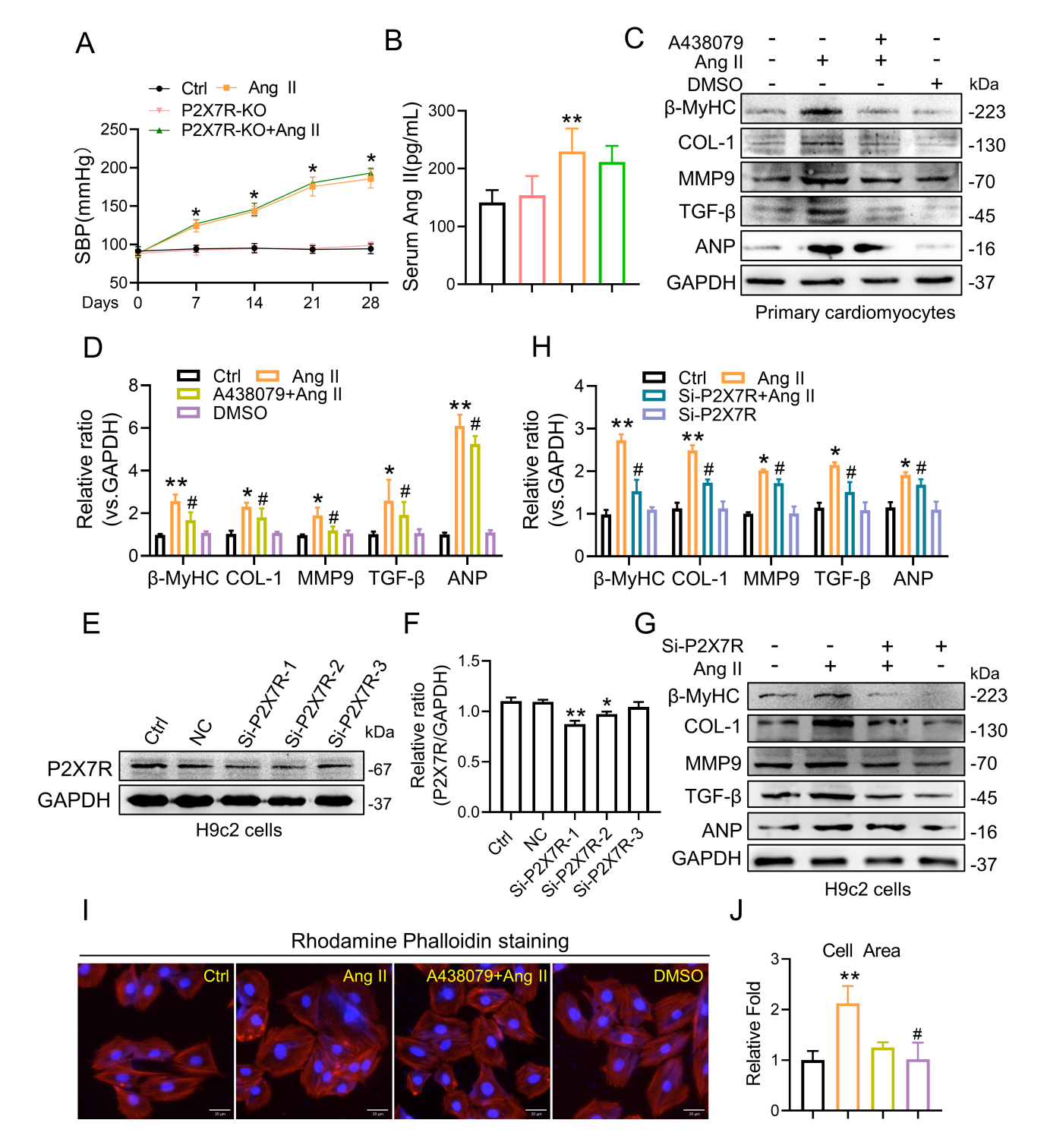

Supplement: Multimedia component 1 [file mmc1.docx]
